# Biological Profile of Two *Gentiana lutea* L. Metabolites Using Computational Approaches and In Vitro Tests

**DOI:** 10.3390/biom11101490

**Published:** 2021-10-09

**Authors:** Simona De Vita, Maria Giovanna Chini, Gabriella Saviano, Claudia Finamore, Carmen Festa, Gianluigi Lauro, Simona De Marino, Roberto Russo, Carmen Avagliano, Agostino Casapullo, Antonio Calignano, Giuseppe Bifulco, Maria Iorizzi

**Affiliations:** 1 Department of Pharmacy, University of Salerno, via Giovanni Paolo II, 132, 84084 Fisciano, Italy; sdevita@unisa.it (S.D.V.); glauro@unisa.it (G.L.); casapullo@unisa.it (A.C.); 2 Department of Biosciences and Territory, University of Molise, Contrada Fonte Lappone, Pesche, 86090 Isernia, Italy; mariagiovanna.chini@unimol.it (M.G.C.); saviano@unimol.it (G.S.); 3 Department of Pharmacy, University of Naples, Via Domenico Montesano, 49, 80131 Naples, Italy; claudia.finamore@unina.it (C.F.); carmen.festa@unina.it (C.F.); sidemari@unina.it (S.D.M.); roberto.russo@unina.it (R.R.); carmen.avagliano@unina.it (C.A.); calignan@unina.it (A.C.)

**Keywords:** natural product, inflammation, computational chemistry, inverse virtual screening, biological profile, inflammation, cancer

## Abstract

Natural products have been the main source of bioactive molecules for centuries. We tested the biological profile of two metabolites extracted from *Gentiana lutea* L. by means of computational techniques and in vitro assays. The two molecules (loganic acid and gentiopicroside) were tested in silico using an innovative technique, named Inverse Virtual Screening (IVS), to highlight putative partners among a panel of proteins involved in inflammation and cancer events. A positive binding with cyclooxygenase-2 (COX-2), alpha-1-antichymotrypsin, and alpha-1-acid glycoprotein emerged from the computational experiments and the outcomes from the promising interaction with COX-2 were confirmed by Western blot, highlighting the reliability of IVS in the field of the natural products.

## 1. Introduction

Natural products (NPs) have always played a major role in the development of new drugs, as reported in a study [1] highlighting that natural products contributed to 65% of the total amount of approved drugs for the treatment of human diseases (accounting data from 1981 to 2014).

Inflammation is a complex and multicomponent pathological pathway that involves a tightly regulated mechanism and several proteins, enzymes, and compounds. In ancient times, to ameliorate the inflammatory state, herbs, administrated usually as infusions, were the only solution. Traditional medicine is undoubtedly one of the oldest and most known “natural” medicines with dozens of medications based on plants and their derivatives. A valuable example is the “Qinjiao”, an anti-inflammatory mixture containing the roots of four plants belonging to the Gentianaceae family (*G. macrophylla*,* G. straminea*,* G. crassicaulis*, and *G. dahurica*) [2]. Another member of the Gentianaceae family, *Gentiana lutea*, has, among other things, considerable anti-inflammatory [2,3,4,5], antiproliferative [6], and antioxidant effects [3]; in particular, the anti-inflammatory effect is mediated by secondary metabolites contained in the roots of this species which down-regulate the expression of cyclooxygenase-2 (COX-2), inducible nitric oxide synthase (iNOS), and pro-inflammatory interleukins [5].

*Gentiana lutea* is a well-known species with a long history of use as herbal medicine in many countries [7], especially for its antiseptic and anti-inflammatory properties [2,8]. The dried underground parts of yellow gentian (i.e., roots and rhizome) are rich in secondary metabolites such as secoiridoids, iridoids, triterpenoids, flavonoids, and xanthones [8]. The classes of secoiridoids and iridoids comprise most of the isolated compounds (gentisin, isogentisin, gentioside, gentiopicroside, gentiopicrin, swertiamarin, sweroside, amarogentin, and loganic acid) considered to be the components responsible for bioactivity [2,9]. In Italy, the infusion of its roots in alcohol produces an extract essential for the preparation of the bitter liqueur “Amaro di Genziana” which is typical of Abruzzo and Alpine regions. The quality of gentian root is evaluated by assessing the major bitter principle, gentiopicroside [10,11]. Antimicrobial [12], antitubercular [13], and wound healing activity [14], gastroprotective [15] and neuritogenic effects [16], and uses as a stimulant of the central nervous system [17] have been reported.

Computational techniques are very useful in the field of natural products (NPs). The identification and characterization of the plant-derived secondary metabolites is generally a challenging task: the extraction and purification steps may be hard or produce an insufficient yield to perform extensive pharmacological profiling on active compounds, their biological partners or mechanism of action may be unknown, and, in some cases, the total synthesis could be difficult or too expensive. In this scenario, a preliminary analysis with computational approaches may orientate the research towards a specific biological pathway, avoiding wasting time, money, and product in unsuccessful tests. Through a structure–activity study, it is also possible to create semi-synthetic analogs of the original molecule, improving its pharmacokinetics/pharmacodynamics properties. Moreover, repurposing campaigns of known NPs may have a double scope: finding new protein partners that can be included in a multi-target pharmacological application and explaining side effects which go beyond the conventional mechanism of action of a particular metabolite. Among the most innovative techniques, Inverse Virtual Screening (IVS) plays a significant role. This methodology, developed and optimized by us in recent years [18,19,20], is designed to retrieve the most probable targets for specific molecule and/or a library of compounds by carrying out molecular docking calculations on a pre-defined panel of protein structures (e.g., belonging to the same pathological pathway, enzyme class, etc.). Several examples have been reported in which IVS was used as the driving force for a repurposing study or in the target identification process, leading to interesting results [20].

**Figure 1:**
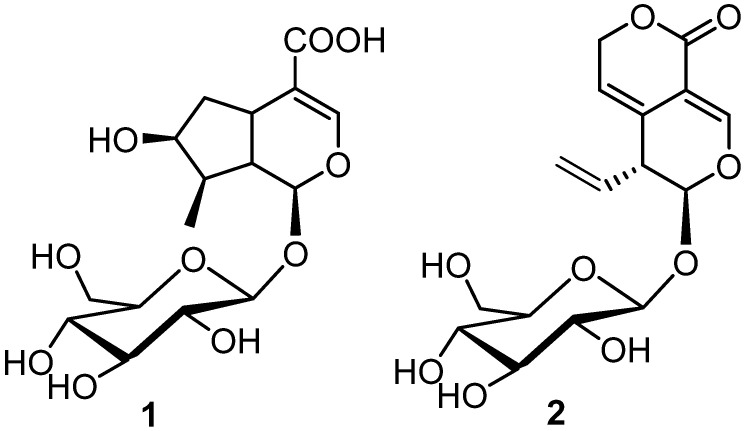
Molecular structure of loganic acid (**1**) and gentiopicroside (**2**).

Based on these considerations, we decided to investigate the anti-inflammatory/anti-cancer properties of loganic acid (**1**) and gentiopicroside (**2**) (Figure 1), two metabolites extracted from *G. lutea*, using a combined approach based on IVS, applied for accomplishing the target identification, and in vitro tests.

## 2. Materials and Methods

### 2.1. General Experimental Procedures

Electrospray ionization mass spectrometry (ESI-MS) experiments were performed on an Applied Biosystem API 2000 triple-quadrupole mass spectrometer. Optical rotations were determined on a Jasko P-2000 polarimeter. Nuclear magnetic resonance (NMR) spectra were recorded on a Bruker Avance NEO 400 spectrometer equipped with an RT-DR-BF/1H-5 mm-OZ SmartProbe (^1^H at 400 MHz and ^13^C at 100 MHz), δ (ppm), *J* in Hz, using a solvent signal for the calibration (^13^CD_3_OD at δ_C_ 49.0 and residual CD_2_HOD at δ_H_ = 3.31). The heteronuclear single quantum coherence (HSQC) spectra were optimized for an average ^1^*J*_CH_ of 140 Hz; the gradient-enhanced heteronuclear multiple bond correlation (HMBC) experiments were optimized for a ^3^*J*_CH_ of 8 Hz. Droplet counter-current chromatography (DCCC) fractionation was performed on DCC-A apparatus (Tokyo Rikakikai Co., Tokyo, Japan) equipped with 250 glass-columns. High-performance liquid chromatography (HPLC) was performed using a Waters 510 pump equipped with a Rheodyne 7125 injector and a Waters 401 differential refractometer as the detector on a C_18_ μ-Bondapak column (30 cm × 3.9 mm i.d) (Waters, Milford, MA, USA).

### 2.2. Plant Material

Selected samples of wild plants *Gentiana lutea* L. (Gentianaceae) were collected in October of 2019 in the Garden of the Apennine Flora of Capracotta (Molise region), a natural botanical garden that preserves the autochthonous flora of the central-southern Apennines in Italy. Plants were identified at the Department of Bioscience and Territory, (University of Molise), and a voucher specimen is deposited under No. GEN-57-2019 in the Herbarium of University of Molise (Pesche, Isernia). Rhizomes were kept frozen at −20 °C until analyzed.

### 2.3. Extraction and Isolation

We followed the extraction method generally used for the preparation of gentian bitter liqueur. The fresh underground parts (167 g) were divided into small pieces for washing and dried at room temperature for 30 days (according to the Italian recipe). Then, rhizomes were extracted in ethanol 96% (3 × 500 mL) at room temperature for 7 days. The combined extracts (16.8 g) were concentrated and subjected to a modified Kupchan’s partitioning procedure [21] to obtain three extracts: *n*-hexane extract (750 mg), CHCl_3_ extract (1.15 g), and *n*-BuOH extract (2.18 g).

The *n*-BuOH extract (2.18 g) was submitted to DCCC with *n*-BuOH/Me_2_CO/H_2_O (3:1:5) in the descending mode (the upper phase was the stationary phase). The obtained fractions were monitored by TLC on Silica gel plates with n-BuOH/OHAc/H_2_O (12:3:5) and CHCl_3_/MeOH/H_2_O (80:18:2) as eluents. Six fractions (A-F) were obtained and purified by HPLC (C18 μ-Bondapak column, 30 cm × 3.9 mm i.d) with MeOH/H_2_O as eluent (25:75; flow rate 1 mL/min). Fraction C (44.3 mg) mainly contained loganic acid while fraction D (73.7 mg; flow rate 0.8 mL/min) afforded gentiopicroside.

^1^H and ^13^C NMR spectra of loganic acid (**1**) and gentiopicroside (**2**) agreed with their previously reported spectroscopic data [22,23].

Loganic acid (**1**). ^1^H NMR (400 MHz, CD_3_OD): δ_H_ 7.39 (1H, s, H-3), 5.26 (1H, br d, *J* = 4.1 Hz, H-1), 4.66 (1H, d, *J* = 7.7 Hz, H-1′), 4.04 (1H, br t, *J* = 4.2 Hz, H-7), 3.89 (1H, d, *J* = 11.8 Hz, H-6′), 3.67 (1H, dd, *J* = 11.8, 3.8 Hz, H-6′), 3.35 (1H, ovl, H-3′), 3.32 (1H, ovl, H-4′), 3.32 (1H, ovl, H-5′), 3.21 (1H, t, *J* = 8.6 Hz, H-2′), 3.09 (1H, m, H-5), 2.23 (1H, m, H-6), 2.03 (1H, m, H-9), 1.85 (1H, m, H-8), 1.65 (1H, m, H-6), 1.09 (3H, d, *J* = 6.8 Hz, H_3_-10). ^13^C NMR (100 MHz, CD_3_OD): δ_C_ 170.1, 151.4, 113.4, 99.2, 96.8, 77.3 (2C), 74.1, 73.8, 70.7, 62.8, 45.7, 41.8, 41.2, 31.3, 12.6. ESI-MS *m*/*z* 375 [M-H]^−^.

Gentiopicroside (**2**). ^1^H NMR (400 MHz, CD_3_OD): δ_H_ 7.44 (1H, s, H-3), 5.75 (1H, m, H-8), 5.66 (1H, d, *J* = 2.8 Hz, H-1), 5.61 (1H, br s, H-6), 5.25 (1H, d, *J* = 13.5 Hz, H-10), 5.21 (1H, d, *J* = 6.8 Hz, H-10), 5.02 (2H, m, H2-7), 4.65 (1H, d, *J* = 7.9 Hz, H-1′), 3.90 (1H, dd, *J* = 11.9, 1.8 Hz, H-6′), 3.66 (1H, dd, *J* = 11.8, 6.2 Hz, H-6′), 3.43 (1H, ovl, H-5′), 3.34 (1H, ovl, H-3′), 3.28 (1H, ovl, H-9), 3.27 (1H, ovl, H-4′), 3.17 (1H, t, *J* = 8.3 Hz, H-2′), ^13^C NMR (100 MHz, CD_3_OD): δ_C_ 168.8, 153.2, 137.5, 129.9, 120.9, 119.6, 107.5, 102.5, 101.0, 80.7, 76.8, 73.9, 73.7, 73.5, 65.1, 49.4. ESI-MS *m*/*z* 355 [M-H]^−^.

### 2.4. Inverse Virtual Screening

In the Inverse Virtual Screening [18,19,20], conversely to classic Virtual Screening, one or few molecules (see Section 2.5) are docked against a large panel of protein targets (cancer- and/or inflammation-related proteins in this case) to highlight putative targets. The target panel accounted in this study contained 3060 proteins that were previously prepared using a tool developed by our group [24] (see Section 2.6).

After molecular docking calculations (see Section 2.7), the binding affinities were collected and normalized. The resulting binding affinities were normalized using a set of 10 decoys to avoid false positive outcomes. Specifically, the decoy molecules share similar chemical features with compounds **1** and **2** (MW, hydrogen bond donor, and hydrogen bond acceptors) but have a chemical structure not related to them. The normalization phase is essential to prevent false-positive results (i.e., a protein target that has a non-specific binding) and it is based on dividing, for each investigated target, the calculated binding affinity for the test compound (*V*_0_) by the average binding affinity value obtained testing decoy molecules (*V_R_*) that share similar chemical features with our compound (Equation (1)). This ratio generates a dimensionless parameter, called “V value”, which is used to obtain a ranking of promising ligand/protein complexes [19,24].

(1) $$V=\frac{V_{0}}{V_{R}}\;$$

### 2.5. Input Molecules Preparation

The input file structures in SDF format were imported in Maestro [25] and processed using the tool LigPrep [26]. In this way, the protonation state of each atom is determined without altering the configuration of the stereogenic centers of the molecule. Afterward, the structures were converted into the PDBQT format using the software OpenBabel v. 2.3.2 (ScienceSoft, McKinney, TX, USA) [27].

### 2.6. Protein Panel Preparation

The crystal structures of the proteins involved in inflammation and cancer were downloaded from the Protein Data Bank (https://www.rcsb.org/, accessed on 25 September 2020) and processed with an automatic workflow recently developed by us [24]. Briefly, the desired protein for the subsequent docking calculations were prepared by removing the unnecessary components in the crystal (solvent molecules, ions, etc.), generating the alternate positions of residues and highlighting the ligands contained, when available. After this preliminary phase, the resulting structures were further processed with the Protein Preparation Wizard tool [28], which was employed to adjust bond orders, to determine the protonation state at physiological pH, and to optimize the intramolecular hydrogen bond network. Each protein structure file in the final panel was then converted in the PDBQT format using the software OpenBabel v. 2.3.2 [27].

### 2.7. Binding Site Detection and Molecular Docking

To correctly drive the grid generation, the binding site of each target must be detected. For this purpose, the software SiteMap (San Jose, CA, USA) [29,30] was used. If in the preparation phase a co-crystallized ligand was detected, its coordinates were used to map the exploration of the surface at a buffer distance of 6Å from it. Otherwise, the whole protein surface was scanned, searching for cavities that could represent a potential binding pocket. In this case, the top five cavities (ranked based on an internal function) were returned, and the best-scoring one was used to build the corresponding molecular docking grid. In both cases, at least 15 site points are required to define a surface as a putative binding site, and the restrictive definition of hydrophobicity was used.

Based on the spatial coordinates of each binding site, the corresponding grid was built around it with a distance buffer of 10 Å in each direction and a spacing of 1.0 Å between the grid points. The molecular docking was carried out with the software AutoDock Vina v. 1.0.2 (The Scripps Research Institute, San Diego, CA, USA) [31] at an exhaustiveness of 64 and treating all open-chain bonds treated as active torsional bonds. Eventually, 10 conformations for each ligand were saved.

### 2.8. Molecular Dynamics

The protein–ligand complexes were prepared for the molecular dynamics simulations with the System Setup tool of the software Desmond v. 6.5 (D. E. Shaw Research, New York, NY, USA) [32,33]. They were inserted in an orthorhombic box, with a buffer distance of 10 Å, and filled with TIP3P water molecules. The system was then neutralized by adding an appropriate amount of Na^+^ and Cl^−^ ions, and a final concentration of 0.15 M of NaCl was added to mimic physiological conditions.

Once ready, the systems were simulated for 100 ns at 310 K in an NPT (namely, keeping constant the number of molecules, pression, and temperature) ensemble using the Nose–Hoover chain as the thermostat method and the Martyna–Tobias–Klein barostat method. At the end of the simulation, the protein–ligand interactions and the total protein RMSD were computed.

### 2.9. In Vitro Experiments

#### 2.9.1. Chemicals

Dulbecco’s modified Eagle’s Medium Ham (DMEM), fetal bovine serum (FBS), and cell culture supplements were purchased from Gibco (Life Technologies, Monza, Italy). Escherichia coli LPS (serotype 0111:B4) was purchased from Fluka (Milan, Italy).

#### 2.9.2. Cell Culture and Treatment

The J774A.1 cell line (BALB/c murine macrophages) was obtained from the European Collection of Animal Cell Cultures (Salisbury, Wiltshire, UK) and cultured in 75 cm^2^ flasks in DMEM supplemented with 10% FBS, 2 mM L-glutamine, 100 U/mL penicillin, 100 μg/mL streptomycin, and 25 mM HEPES at 37 °C under 5% CO_2_ humidified air.

Cells were mechanically scraped and plated (400,000 cells/p60 dish). After 4 h to allow adhesion, cells were starved in 5% FBS for 2 h and subsequently pre-treated for 1 h with **1** and **2** (1, 3, 10 µM). Then, cells were incubated with LPS (100 ng/mL) for 24 h. The final concentration of ethanol in control and treated cells was 1% (*v*/*v*).

#### 2.9.3. Western Blot Analysis

For the preparation of whole-cell lysates, cells were washed with ice-cold phosphate-buffered saline (PBS), harvested, and resuspended in lysis buffer (20 mM Tris–HCl (pH 7.5), 10 mM NaF, 150 mM NaCl, 1% Nonidet P-40, 1 mM phenylmethylsulfonyl fluoride, 1 mM Na_3_VO_4_, leupeptin, and trypsin inhibitor 10 μg/mL). After 1 h, cell lysates were obtained by centrifugation at 20,000× *g* for 15 min at 4 °C. Protein concentrations were estimated by a Bio-Rad protein assay using bovine serum albumin (Sigma-Aldrich, Milan, Italy) as standard. For Western blot analysis, protein cell lysates (30 μg) were dissolved in Laemmli sample buffer, boiled for 5 min, and subjected to SDS-polyacrylamide gel electrophoresis and transferred to the nitrocellulose membrane (Amersham Biosciences, Little Chalfont, Buckinghamshire, UK) using a Bio-Rad Transblot (Bio-Rad, Milan, Italy). The filter was blocked with 1× phosphate-buffered saline (PBS) and 3% non-fat dried milk at room temperature and probed with anti-cyclooxygenase (COX)-2 (dilution 1:1000; cat. no. 610204, BD Bioscience, from Becton Dickinson, Buccinasco, Italy), or anti-inducible nitric oxide synthase (iNOS) antibody (dilution 1:1000; cat. no. 610432, BD Bioscience) in 1 × PBS, 3% non-fat dried milk, and 0.1% Tween 20 at 4 °C overnight. The secondary antibody (Jackson ImmunoResearch Laboratories, Baltimore Pike, West Grove, PA, USA) was incubated for 1 h at room temperature. Then, the blot was extensively washed with PBS, developed using enhanced chemiluminescence detection reagents (Amersham Pharmacia Biotech, Piscataway, NJ, USA) according to the manufacturer’s instructions, and the immune complex was visualized by Image Quant (GE Healthcare, Milan, Italy). The protein bands were densitometrically analyzed with a model GS-700 imaging densitometer (Bio-Rad Laboratories, Milan, Italy). To ascertain that the blots were loaded with equal amounts of protein lysates, they were also incubated in the presence of the antibody against the β-actin protein (cat. no. A5441, Sigma-Aldrich, Milan, Italy).

### 2.10. Statistical Analysis

Data are presented as mean ± SEM. Statistical analysis was performed by analysis of variance test for multiple comparisons followed by Bonferroni’s test, using GraphPad Prism (GraphPad Software, San Diego, CA, USA). Statistical significance was set at *p* < 0.05.

## 3. Results

In this work, the anti-inflammatory/anti-cancer properties of two secondary metabolites, loganic acid (**1**) and gentiopicroside (**2**), extracted from *G. lutea*, were elucidated using both in silico and in vitro methodologies. After the extraction and characterization step, a multistep computational protocol was applied to single out the potential protein partner(s) for **1** and **2**.

As reported above, the IVS approach becomes very handy when treating NPs; thus, this methodology was here used to highlight the most probable binding partners among a large pool of proteins involved in inflammation and cancer. Afterwards, in order to validate the outcomes obtained from molecular docking studies, the stability of NP/target complexes was assessed by performing molecular dynamics simulations to monitor the binding trend of each of them. The results were eventually corroborated by Western Blot analysis.

### 3.1. Inverse Virtual Screening

The Inverse Virtual Screening (IVS) computational approach was used in this study to predict the most promising interacting targets of compounds **1** and **2** by testing them against a panel of proteins (3060 items) involved in cancer or inflammation.

In detail, the identification of the most promising putative biological macromolecule counterparts was accomplished following three different phases: (1) molecular docking calculation of the two small molecules towards the protein panel; (2) the normalization of the binding affinities related to each obtained metabolite/protein complex; (3) computational analysis of the selected ligand/protein systems.

Firstly, the two compounds (**1** and **2**) were screened against a panel containing proteins by molecular docking calculations. Then, the normalization step, which is a mathematical manipulation of the binding affinities from docking calculations to determine the peculiar trend for each compound against every single target, was performed. For this purpose, the corresponding binding affinities were normalized using 10 “decoy” molecules, which are compounds showing chemical features and molecular weight properties similar to compounds **1** and **2** but different chemical structures (see Section 2.7 for further details). The use of decoys is particularly useful to avoid the systematic selection of false positive results and, contrariwise, the exclusion of false negatives.

From the normalization step, a dimensionless number *V* is calculated and applied to rank the most promising interacting macromolecules counterparts.

In our analysis, only the proteins with a *V* value above 0.9 were kept and analyzed. In detail, Table 1 and Table 2 contain the top 10 non-redundant targets (since the protocol used for the generation of the panel [24] may produce multiple conformations for the same protein) for the two molecules **1** and **2** and the occurrences of specific type of target on the normalized and filtered panel.

Interestingly, some targets were identified for both the two compounds (**1** and **2**). Specifically, alpha-1-antichymotrypsin, prostaglandin H_2_ Synthase-2, also known as COX-2, and alpha-1-acid glycoprotein 2, which are known to be involved in both pathological pathways that are the focus of our studies [34,35,36,37,38,39], represented interesting results. For these reasons, the following analysis was focused on the targets in common to rationalize the anti-inflammatory properties reported for both compounds. Therefore, a detailed exposition of each protein/ligand complex is reported below that aims to reveal, at molecular level, the binding mode of each compound with its protein counterpart. This type of analysis will help to discriminate whether the selected targets are suitable candidates to rationalize the biological effects of **1** and **2**.

#### 3.1.1. Alpha-1-antichymotrypsin

**Figure 2:**
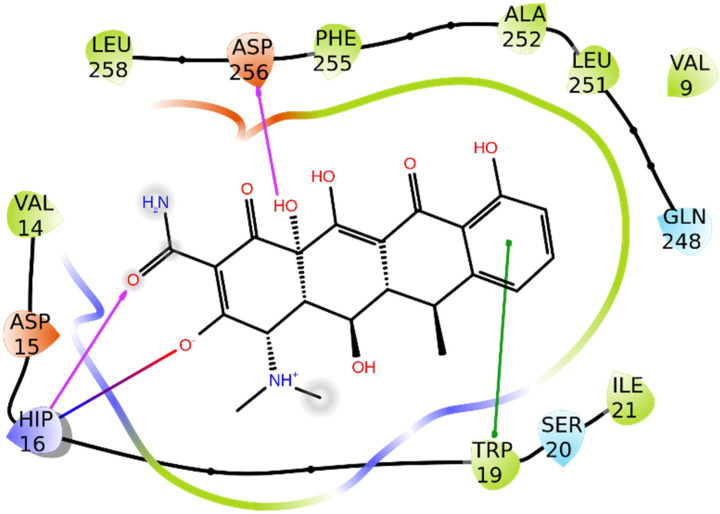
Binding mode of doxycycline with alpha-1-antichymotrypsin (5OM2) [41]. Pink arrows are hydrogen bonds, the green line represents a π–π interaction, and the red-to-blue line represents an ionic bridge. Green residues are hydrophobic ones, cyan indicates polar residues, and red or blue ones are negatively and positively charged, respectively.

This protein belongs to a family of serpins, which are structurally characterized by three β sheets and several α helices [40]. Serpins have protease activity and are involved in pathological states such as inflammation or fibrinolysis. According to both what was previously reported and the interactions made by the co-crystallized molecule of doxycycline (PDB code: 5OM2) (Figure 2), His16, Trp19, and Asp256 were identified as the most interesting residues. Moreover, the binding pocket contains many hydrophobic residues, which are ideal for accommodating a molecule such as doxycycline.

**Figure 3:**
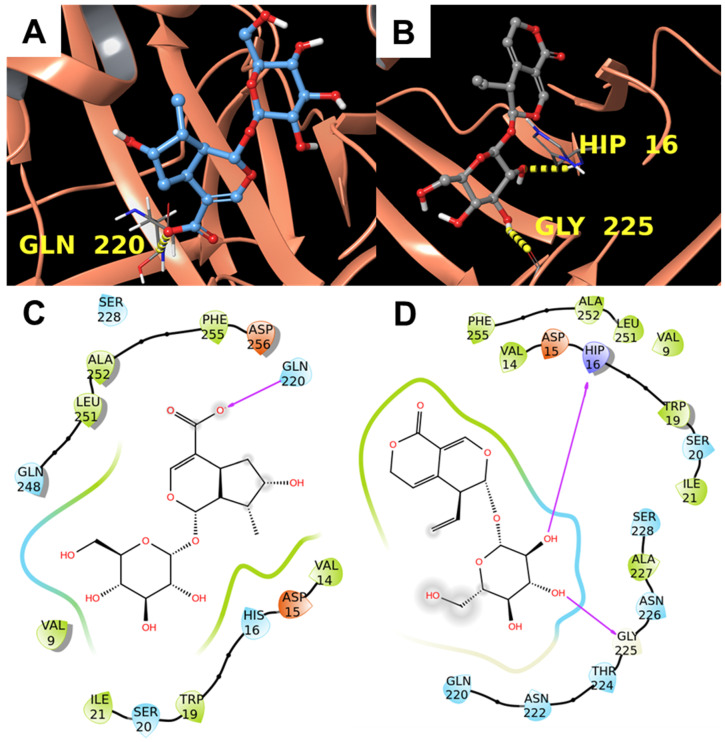
Molecular docking results. (**A**,**B**) 3D binding mode of **1** (**A**) and **2** (**B**) with alpha-1-antichymotrypsin (PDB: 5OM2 [41], orange ribbons). Hydrogen bonds are represented by yellow dotted lines. (**C**,**D**) 2D representation of the binding pose of **1** (**C**) and **2** (**D**). Pink arrows represent hydrogen bonds.

The visual inspection of the calculated docking poses of **1** and **2** suggested a possible interaction with the target. In detail, **1** formed a hydrogen bond with the backbone of Gln220, and **2** interact with His16 and Gly225 through hydrogen bonds made by the sugar ring (Figure 3).

**Figure 4:**
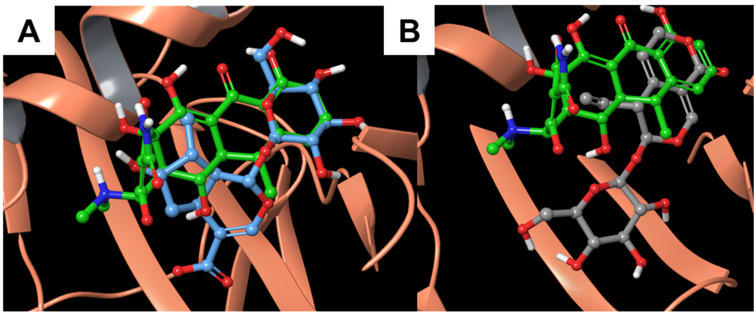
Superimposition between the co-crystallized doxycycline and (**A**) **1** and (**B**) **2** inside the alpha-1-antichymotrypsin binding site (PDB: 5OM2 [41], orange ribbons).

Moreover, the superimposition between our compounds and the co-crystallized doxycycline (Figure 4) indicated that both molecules are oriented like the known ligand, especially the ring moieties, suggesting a discrete degree of interaction, especially for compound **1**, between gentian-derived molecules and alpha-1-antichymotrypsin.

#### 3.1.2. COX-2

**Figure 5:**
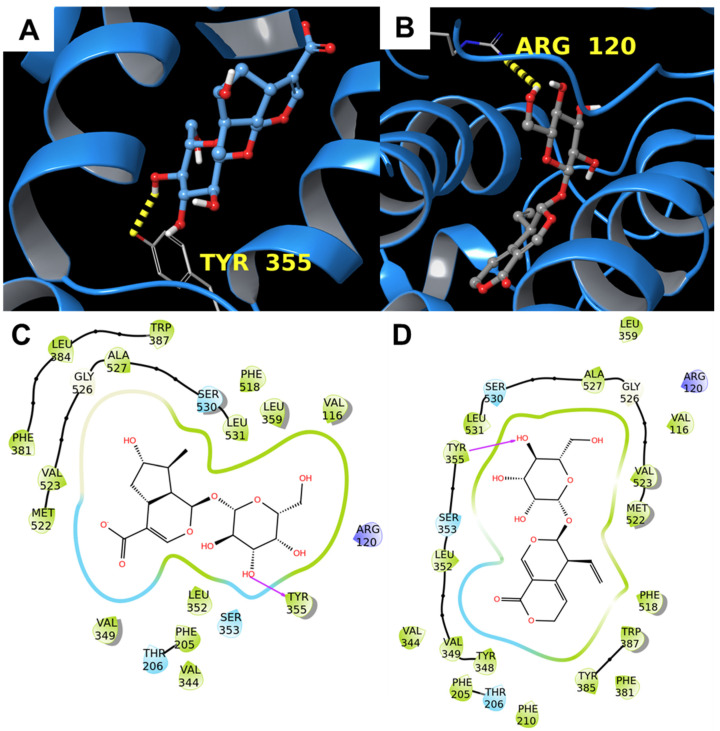
Molecular docking results. (**A**,**B**) 3D binding mode of **1** (**A**) and **2** (**B**) with COX-2 (PDB: 1DDX [44], cyan ribbons). Hydrogen bonds are represented by yellow dotted lines. (**C**,**D**) 2D representation of the binding pose of **1** (**C**) and **2** (**D**). Pink arrows represent hydrogen bonds.

The prostaglandin H_2_ synthase-2 (also known as COX-2) is a key enzyme involved in acute inflammation and it contains three different domains: an EGF domain, a membrane-binding domain characterized by α helices, and a catalytic domain that converts arachidonic acid into prostaglandin H_2_. The binding site of COX-2 consists of a catalytic triad made by Arg120, Tyr355, and Glu524 [42]; additional residues involved in COX-2 inhibition are Tyr385 and Arg513 [43]. Both compounds were well inserted in the binding pocket and interacted with one of the catalytic residues (Tyr355 and Arg120 for **1** and **2,** respectively) through the glycosyl moiety (Figure 5). As already mentioned before, the calculated binding affinities (−9.5 kcal/mol for both compounds) provided a further clue that COX-2 may be a privileged target for **1** and **2**.

**Figure 6:**
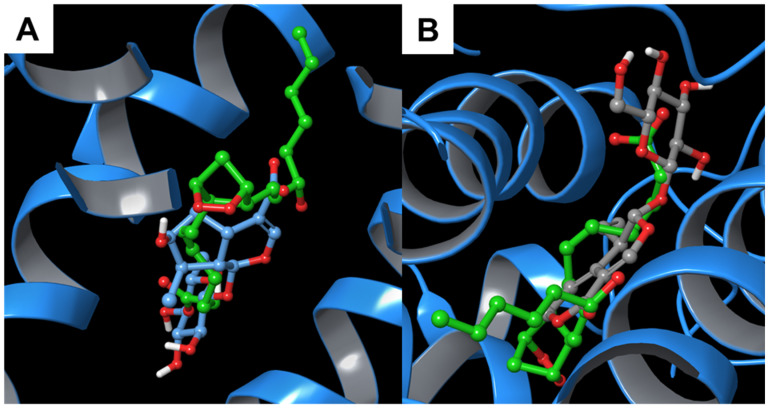
Superimposition between the co-crystallized prostaglandin G_2_ and (**A**) **1** and (**B**) **2** inside the binding pocket of COX-2 2 (PDB: 1DDX [44], cyan ribbons).

In addition, the superimposition with the co-crystallized residue showed that the compounds of interest were well aligned with the three-dimensional arrangement of the referring molecule (Figure 6).

#### 3.1.3. Alpha-1-Acid Glycoprotein

**Figure 7:**
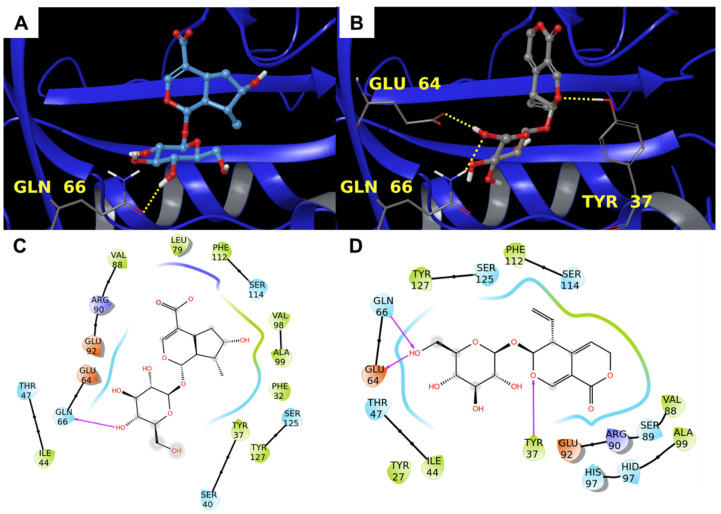
Molecular docking results. (**A**,**B**) 3D binding mode of **1** (**A**) and **2** (**B**) with alpha-1-acid glycoprotein (PDB: 3APW [47], blue ribbons). Hydrogen bonds are represented by yellow dotted lines. (**C**,**D**) 2D representation of the binding pose of **1** (**C**) and **2** (**D**). Pink arrows represent hydrogen bonds.

The alpha-1-acid glycoprotein is a plasmatic protein synthesized by the liver that is known to have a pro-inflammatory effect [45]. The specific function of this protein is still not clear, but it seems to act as a steroid carrier, inhibit the neutrophil chemotaxis, inhibit platelet aggregation, and influence immune system proliferation [46]. Moreover, the expression of alpha-1-acid glycoprotein is regulated by peroxisome proliferator–activator receptor gamma (PPAR-γ), which is well known for being a key anti-inflammatory component. From what was reported [47,48], among the required interactions, there is a polar moiety constituted by Tyr37, Ser40, Gln66, Arg68, Arg90, Glu92, Ser114, Ser125, and Tyr127. The two compounds made positive contact with this hydrophilic surface because of their chemical structure. In detail, **1** established a hydrogen bond with Gln66 and **2** interacted via hydrogen bonds with Tyr37, Glu64, and Gln66 (Figure 7).

**Figure 8:**
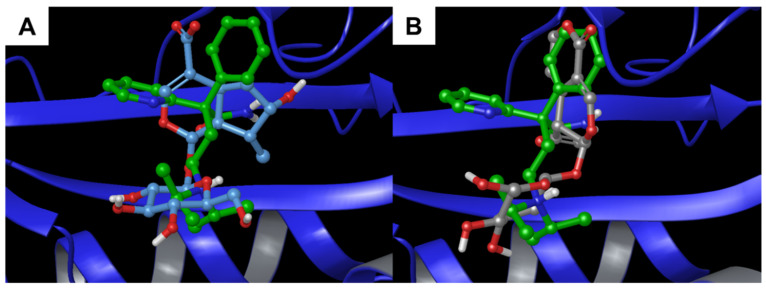
Superimposition between the co-crystallized prostaglandin G_2_ and (**A**) **1** and (**B**) **2** inside the binding pocket of with alpha-1-acid glycoprotein (PDB: 3APW [47], blue ribbons).

Additionally, in this case, the comparison with the co-crystallized ligand showed a positive superimposition with the two natural products (Figure 8). Particularly, the bicyclic moiety of **1** and **2** were orientated towards the pyridyl and the phenyl group, respectively.

### 3.2. Molecular Dynamics

In order to corroborate the IVS results, extensive molecular dynamics (MD) simulations (100 ns) were performed in explicit solvent (Desmond software [32,33]) to capture the dynamic nature of protein–ligand interactions.

In particular, for analysis of the trajectories, the simulation interactions diagram (SID) (Maestro version 10.2) was used as a tool for exploring protein–ligand interactions to assess whether the connections highlighted in the molecular docking were kept during the simulation. In this way, it is possible to estimate the entity of the binding and detect the residues most involved in the ligand/protein interaction. With this analysis, it is also possible to determine whether the functional amino acids of each binding site interact with the compound under evaluation and, accordingly, an interference with the protein activity may be hypothesized.

**Figure 9:**
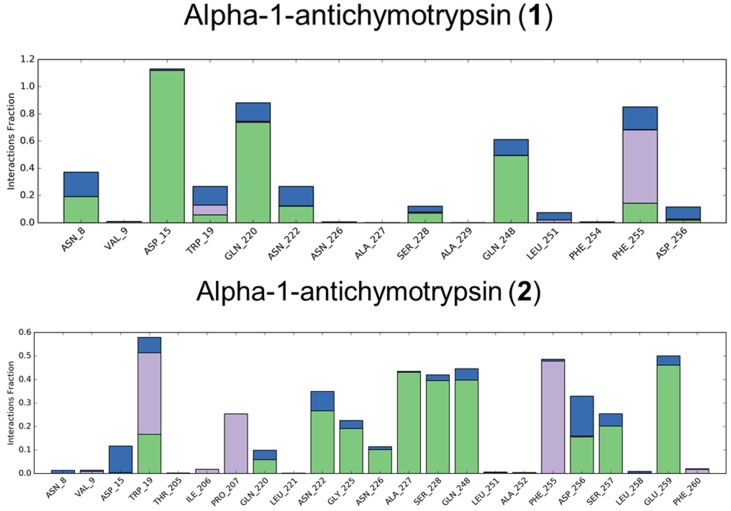

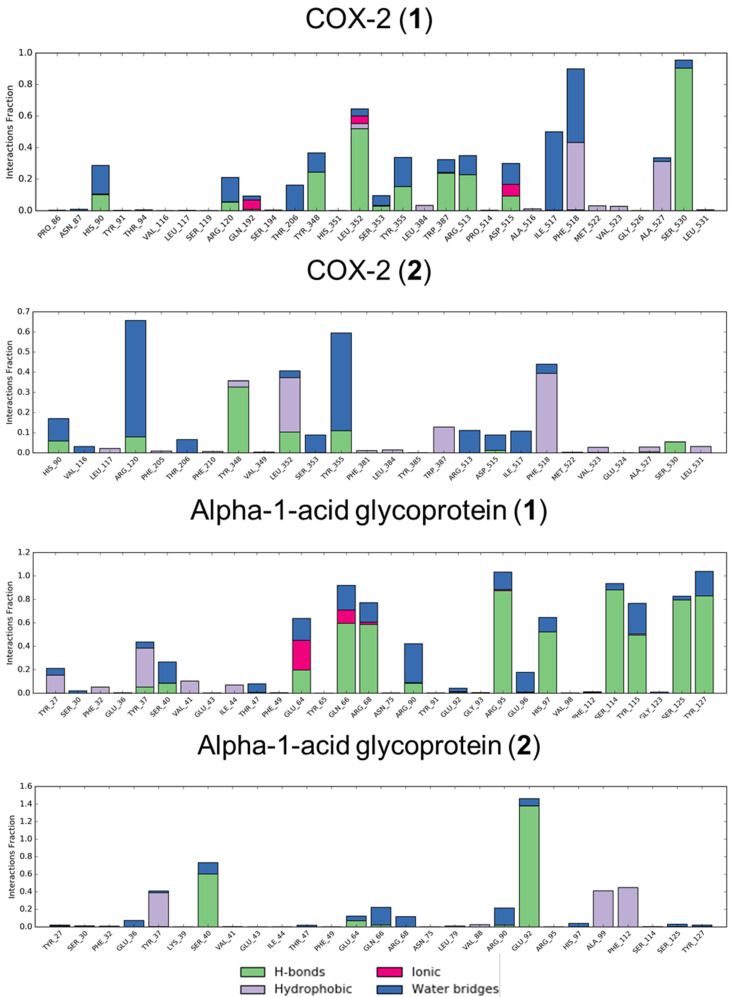
Protein–ligand interactions made by the six complexes during the 100 ns molecular dynamics simulation. The entity of the binding is expressed as fraction of the total simulation time and the different contributions are listed with different colors.

From the data reported in Figure 9, it is noteworthy that the main interacting residues obtained by the molecular dynamics simulations corresponded to those suggested by the molecular docking experiments for both the secondary metabolites. Specifically, the encouraging interactions pattern and the calculated binding affinity reported for COX-2 were reflected in the extensive number of contacts made. In particular, compound **1** interacted stably with Ser530, which is a pivotal residue in the inhibition of this enzyme [49], reaching almost 100% of the time. The other two protein targets showed good results, but one of the two small molecules performed slightly better than the other one (compound **2** for alpha-1-anthichymotrypsin and compound **1** for alpha-1-acid-glycoprotein, respectively).

In addition, to corroborate the results above, the RMSD of the protein was monitored during the simulation (Supporting Information). Small variations in the RMSD, in fact, indicated small variation in the protein structure reflecting a good stabilization of the ligand/target complex.

One of the most interesting consideration deducible from the analysis of the RMSD trend (Figure S3) is that the complexes remained stable throughout the 100 ns of MD simulation and that the fluctuations recorded were never above 3.5 Å, confirming the entity of the interaction between each protein and the small molecule bound to it.

In summary, even if the specific patterns of hydrogen bonds and the number of hydrophobic contacts between **1** and **2** with the three selected macromolecules seemed to be the driving forces of the target–ligand complexes, from the detailed MD analyses reported above, COX-2 appeared as the most promising interacting target and, therefore, it was chosen as the focus for the in vitro tests.

### 3.3. In Vitro Analysis

#### Effect of **1** and **2** on COX-2 and iNOS Expression in J774A.1 Cells

The concentrations of both substances for in vitro tests were chosen because they did not significantly modify J774A.1 cell viability (data not shown).

**Figure 10:**
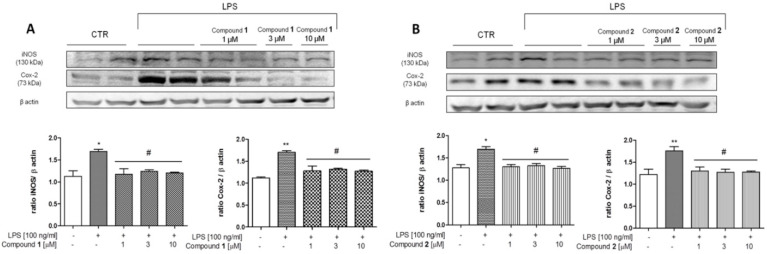
Effect of (**A**) **1** and (**B**) **2** on COX-2 expression in J774A.1 cells. Lysates were obtained from control cells and 24 h-treated cells with both compounds at increasing concentrations (1–3–10 µM) after 24 h LPS (100 ng/mL) treatment. A representative immunoblot is shown. Densitometric analysis of protein bands was performed on three separate experiments. * *p* < 0.05 and ** *p* < 0.01 vs. control cells; # *p* < 0.05 vs. LPS-treated cells. “+ (presence), − (absence)” of LPS or of the compounds under study.

COX-2 and iNOS expression was evaluated to determine the modulatory effect of **1** or **2** in combination with LPS on the induction of these pro-inflammatory enzymes. As reported in Figure 10, LPS (100 ng/mL) in J774A.1 cells for 24 h significantly increased iNOS and COX-2 expression with respect to CTR (** *p* < 0.01 and * *p* < 0.05 vs. CTR). When the cells were pre-stimulated for 1 h with **1** (1, 3, 10 µM) and **2** (1, 3, 10 µM), a significant reduction in COX-2 and iNOS expression was observed (# *p* < 0.05 vs. LPS, Figure 10A,B).

## 4. Discussion

In this study, the biological activities displayed by two compounds extracted from *G.lutea*, namely loganic acid (**1**) and gentiopicroside (**2**), were rationalized at the molecular level. These two compounds were extracted from the plants collected in the Garden of the Apennine Flora of Capracotta and characterized using NMR and mass spectroscopy. In order to assess the pharmacological partners of **1** and **2**, an in silico multistep protocol involving Inverse Virtual Screening and molecular dynamics simulations was used. The IVS approach, in fact, is very useful to single out and suggest putative macromolecule counterparts for molecules with unknown interacting proteins, and for these reasons, it represents an interesting strategy for drug repositioning or target identification of bioactive natural and/or synthetic compounds. In detail, the IVS procedure was applied to identify the most probable protein partners for each compound using molecular docking calculations. Then, the predicted binding affinities computed in the previous step were normalized (see above) by dividing the calculated binding affinity of the compound against a specific target by the average affinity of 10 “decoy” molecules on the same target. In this way, a dimensionless number, the *V* parameter, is obtained and used to indicate an encouraging interaction between the compound of interest and the protein. In the final phase of IVS, the protein/ligand complexes are visually inspected and evaluated in terms of energy and key interactions made for the binding of the compound under study with the protein partner. It was interesting to notice that several top-ranked proteins were common between **1** and **2**; in particular, alpha-1-antichymotrypsin, prostaglandin H_2_ synthase (COX-2), and alpha-1-acid glycoprotein resulted as the most promising ones. All three macromolecules are known to be both key factors in acute inflammation and involved in the progression of cancer as biomarkers [36,37,38,39] or as effective players [34,35]. After an extensive investigation, using the IVS approach on a panel containing inflammation and cancer proteins, the two molecules (**1** and **2)** were proved to interact with the crucial amino acids of each protein’s binding site and show a high degree of superimposition with the co-crystallized ligand contained in each pocket, indicating a putative interaction with the selected targets. Starting from the IVS results, we decided to improve and confirm the initial hypothesis by performing a molecular dynamics (100 ns) of each protein/ligand complex to assess, at the molecular level, whether the intermolecular contacts highlighted in the previous step are kept or not. Molecular dynamics allows a deeper investigation into the nature of each interaction and was very useful to discriminate the most promising binder. From the analysis of the protein/ligand contacts combined with the RMSD values, it emerged that all the systems are stable over time thanks to an extended hydrogen and hydrophobic contact network. The predicted interaction with COX-2, which is the source of the acute phase inflammation mediators, was of particular interest. Considering the optimal predicted binding affinity and interaction pattern with key amino acids shown by both compounds, the effect of the treatment with **1** and **2** was assessed in vitro with Western blot analysis. The macrophagic cell line J774A.1 was used because the capacity to respond to a variety of stimulating agents, including bacterial LPS and inflammatory mediators, represents an important property of the monocyte–macrophage. In particular, the expression of the main two pro-inflammatory enzymes, such as COX-2 and iNOS, was evaluated. Our data pointed out a protective and significant effect of both compounds, **1** and **2**, even to the lowest dose of 1 µM, suggesting a strong anti-inflammatory activity in vitro.

## 5. Conclusions

With the aim of elucidating the molecular mechanisms of the anti-inflammatory effects of *Gentiana lutea*, two metabolites, loganic acid (**1**) and gentiopicroside (**2**), were evaluated in silico combining a robust technique called Inverse Virtual Screening and molecular dynamics simulations. In more detail, the IVS approach is useful for predicting the most promising interacting targets of the investigated compounds using a large panel of proteins related to a specific pathology or biological pathway, such as inflammation. In this specific case, for the IVS step, we have customized the panel proteins considering all the protein available in PDB involved in the inflammation and cancer events. After the molecular docking calculations and the normalization step (see above), the V values better than 0.9 were used to select the most promising interacting macromolecules counterparts. Comparing the selected targets able to interact with both the secondary metabolites, three putative candidates (alpha-1-antichymotrypsin, prostaglandin H_2_ synthase (COX-2), and alpha-1-acid glycoprotein) were initially selected due to their particularly favorable binding and spatial disposition in the target binding site. After this step, thanks to a molecular dynamics simulation in an explicit solvent, COX-2 was identified as the most promising target in which the protein/ligand interactions and the total protein RMSD was recorded during the 100 ns.

These in silico predictions supported in vitro results, obtained using a macrophage cell line (J774A.1) because of its capability of reacting to pro-inflammatory stimuli. From the Western blot analysis, the expression of COX-2 was visibly reduced, sustaining the predictions obtained in the computational step of this study. Summarizing all the outcomes reported in this work, the biological studies are in line with IVS results, highlighting the efficiency of this method in accomplishing the target prediction task. Accordingly, this approach can be used to both identify the most likely macromolecule counterpart for NPs and disclose new applications for those NPs with a known biological profile, reducing time and costs of the development phase. The in silico/in vitro combined approach is, therefore, highly recommended for the rapid assessment of the biological profile of bioactive secondary metabolites.

## Figures and Tables

**Table 1:** IVS target ranking for **1**. The percentage of occurrences is calculated on the number of target structures with a *V* value above 0.9.

Target	PDB	*V* *	Calculated Binding Affinity (*V*_0_)	Average Decoy Binding Affinity (*V*_R_)	% of Occurrences
Transient Receptor Potential Cation Channel Subfamily V Member 1	5irz	1.48	−7.7	−5.2	0.3%
Alpha-1-Acid Glycoprotein 2	3apw	1.18	−8.3	−7.0	0.1%
Transient Receptor Potential Cation Channel Subfamily V Member 1	5is0	1.18	−8.7	−7.3	0.3%
Tumor Necrosis Factor Ligand Superfamily Member 4	2hew	1.13	−9.4	−8.3	0.2%
Complement C3 Membrane Cofactor Protein	5fo8	1.12	−9.5	−8.5	2.6%
Alpha-1-Antichymotrypsin	5om2	1.12	−8.6	−7.7	1.2%
Tumor Necrosis Factor Ligand Superfamily Member 4	2hey	1.11	−9.2	−8.2	0.2%
Alpha-1-Acid Glycoprotein 2	3apu	1.10	−9.1	−8.3	0.1%
Prothrombin	4ax9	1.10	−7.8	−7.1	23.3%
Prostaglandin H**_2_** Synthase-2	1ddx	1.09	−9.5	−8.7	0.6%

* *V* = *V*_0_/*V*_R_.

**Table 2:** IVS target ranking for **2**. The percentage of occurrences is calculated on the number of target structures with a *V* value above 0.9.

Target	PDB	*V* *	Calculated Binding Affinity (*V*_0_)	Average Decoy Binding Affinity (*V*_R_)	% of Occurrences
Alpha-1-Acid Glycoprotein 2	3apw	1.21	−8.5	−7.0	0.1%
Alpha-1-Antichymotrypsin	5om2	1.13	−8.7	−7.7	1.2%
Alpha-1-Antichymotrypsin	5om3	1.10	−8.6	−7.8	1.2%
Prostaglandin H2 Synthase-2	1ddx	1.09	−9.5	−8.7	1.0%
Tumor Necrosis Factor Ligand Superfamily member 4	2hew	1.09	−9.0	−8.3	0.1%
Alpha-1-Antichymotrypsin	5om7	1.08	−8.3	−7.7	1.2%
Tyrosine-Protein Kinase BTK	6aub	1.07	−8.0	−7.4	3.2%
Alpha-1-Antichymotrypsin	5om7	1.06	−8.3	−7.8	1.2%
Tyrosine-Protein Kinase BTK	6bik	1.06	-8.1	−7.6	3.2%
Tumor Necrosis Factor Ligand Superfamily Member 4	2hey	1.05	−8.7	−8.28	0.1%

* *V* = *V*_0_/*V*_R_.

## Data Availability

Not applicable.

## Supplementary Materials

The following are available online at https://www.mdpi.com/article/10.3390/biom11101490/s1, Figure S1: ^1^H NMR spectra of loganic acid (**1**) (400 MHz, CD3OD), Figure S2: ^1^H NMR of genti-opicroside (**2**) (400 MHz, CD3OD), Figure S3: RMSD trend for the six complexes during the 100 ns MD simulation.
