# Supplementary material for: Biological Profile of Two Gentiana lutea L. Metabolites Using Computational Approaches and In Vitro Tests
Source: Biomolecules. 2021 Oct 9;11(10):1490. doi: 10.3390/biom11101490 (PMC8533323; doi:10.3390/biom11101490)
Supplement: Supplementary file 1 [file biomolecules-11-01490-s001.zip › biomolecules-1390910-supplementary.pdf]

Supplementary

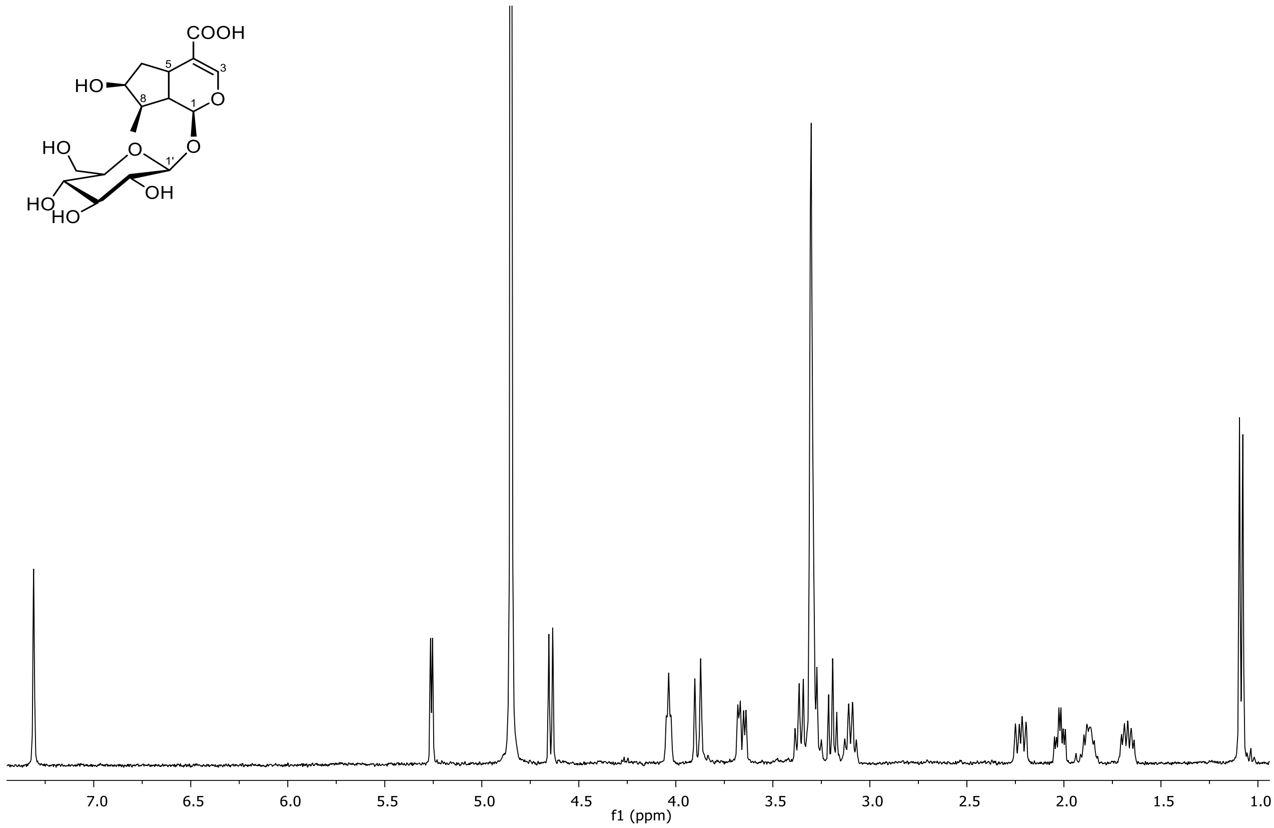

Figure S1. ^{1}H NMR spectra of loganic acid (**1**) (400 MHz, CD3OD).

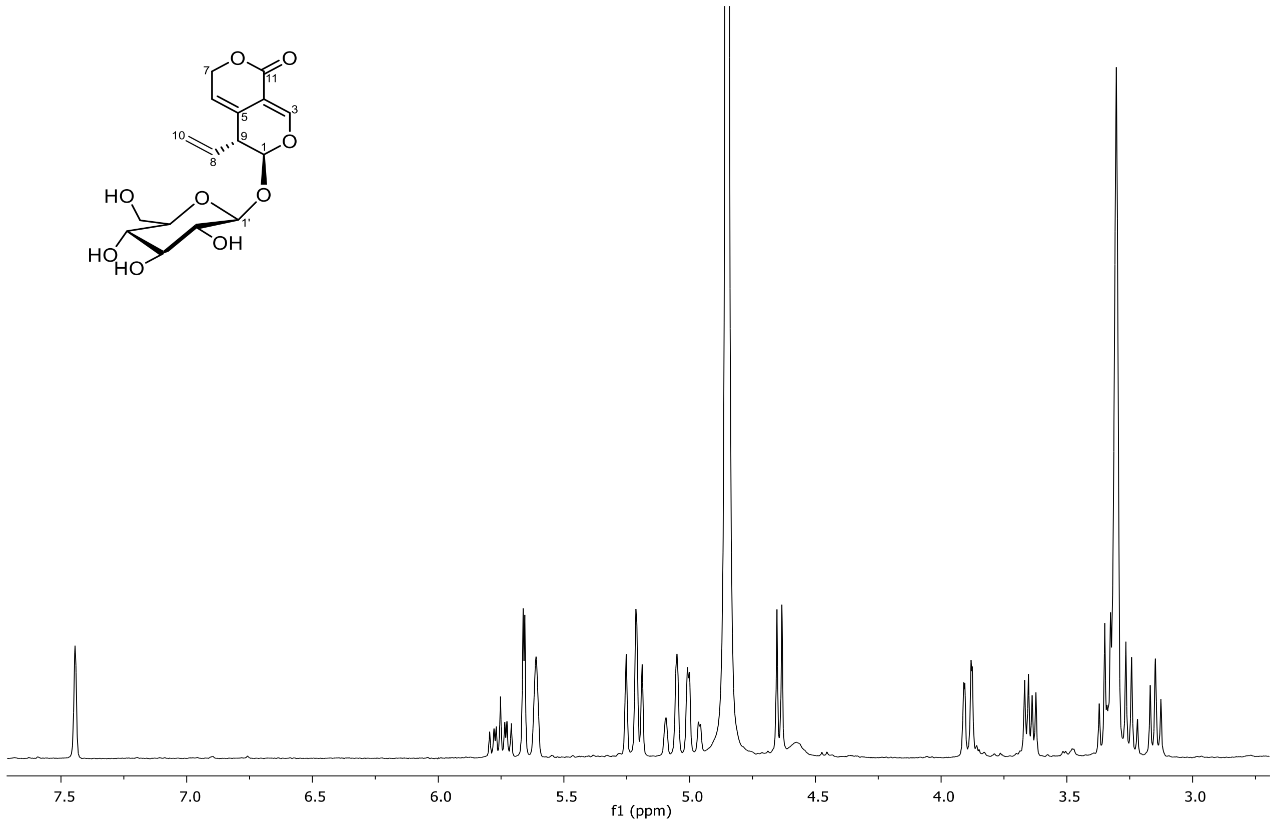

Figure S2. ^{1}H NMR of gentiopicroside (**2**) (400 MHz, CD3OD).

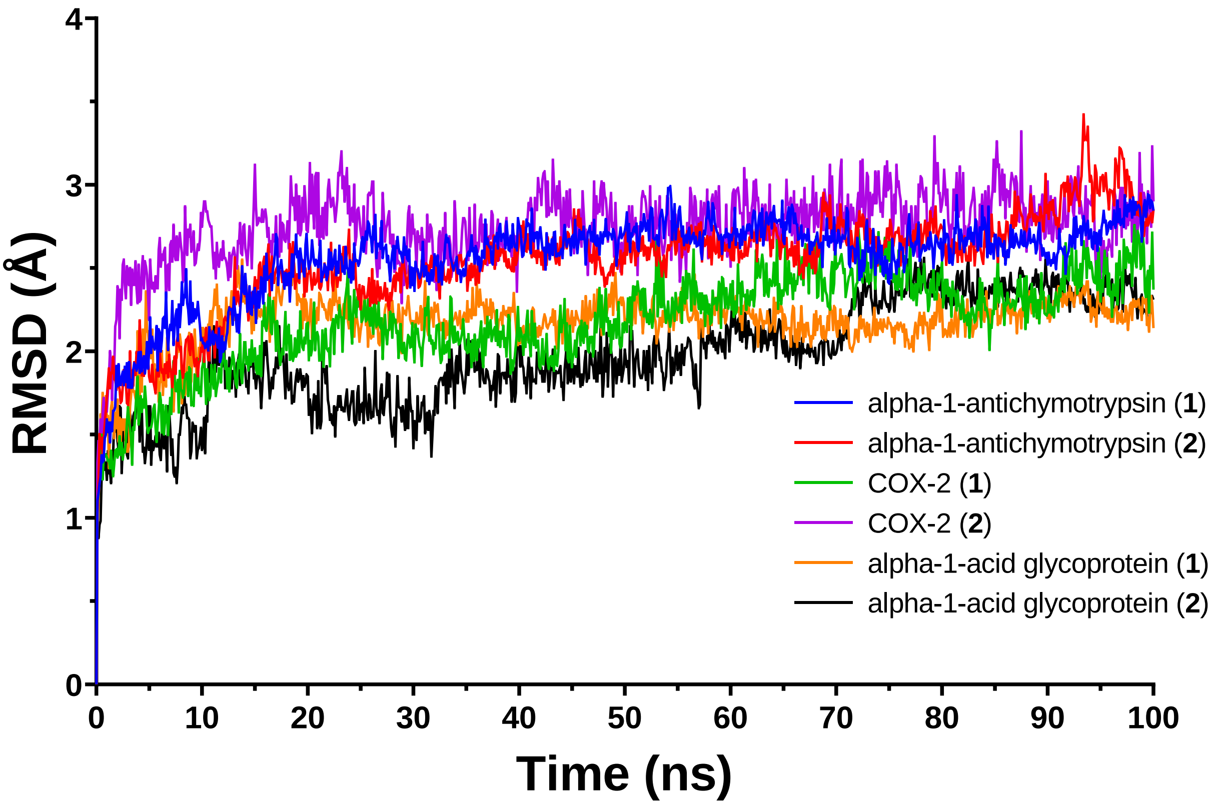

Figure S3. RMSD trend for the six complexes during the 100 ns MD simulation.
